# Trajectories of Successful Aging Among Older Adults in Aotearoa New Zealand

**DOI:** 10.1177/07334648231163052

**Published:** 2023-03-14

**Authors:** Ayodeji Fasoro, Wendy Maddocks, Pauline Barnett, Arindam Basu

**Affiliations:** 1 Faculty of Health, 2496 University of Canterbury, Christchurch, New Zealand

**Keywords:** successful aging, longitudinal methods, population aging, older adults, latent growth curve modeling, New Zealand

## Abstract

Successful aging was defined as having no multimorbidity, high functional capacity, active life engagement, and good health-related quality of life. This study analyzed data from 1433 older adults who were followed up for 12 years across seven waves from the New Zealand Health, Work and Retirement study by examining the trajectories of successful aging. Latent growth curve modeling was used to assess the growth factors of successful aging trajectories of older adults. The mean successful aging score was 3.53 (range: 0–6) in 2006 and linearly declined by 0.064 units every year. Those with higher successful aging scores at baseline had a slower decline. Successful aging scores were lower among females, Māori, and those aged 65 years and above at baseline. The findings from this study suggest that gender and ethnic inequalities play significant roles in successful aging among older adults in New Zealand.

**What this paper adds**

- Successful aging is on a continuum and is best understood through longitudinal studies.
- Multimorbidity, functional capacity, life engagement, and health-related quality of life are important determinants of successful aging.
- Age, gender, and ethnicity are predictors of successful aging latent intercept, but only age predicts the latent slope.

**Applications of study findings**

- Culturally appropriate models are needed to assess successful aging among indigenous people.
- Addressing health inequalities among ethnic groups will improve how older adults age well.

## Introduction

All people age and different people age differently. Rowe and Kahn (1987) developed a model of “successful aging” based on a longitudinal study of high-functioning, disability-free 70–79-year-old older adults in the United States. According to this model, successful aging is predicated on a low likelihood of disease and disease-related disability, high cognitive and physical functional capacity, and active engagement with life (Rowe & Kahn, 1987). Researchers have critiqued the model and the successful aging terminology (Dillaway & Byrnes, 2009) on the basis that the model is not sufficiently inclusive of factors that promote aging well. We also argue that this model has three limitations worth considering. First, the presence of disease and disability does not necessarily mean poor health-related quality of life, and quality of life is an important consideration in older people’s health care in order to ensure their health and overall wellbeing rather than only management of the disease (Lee et al., 2022; Teovska Mitrevska et al., 2012). Second, the model is not sufficiently inclusive of factors that promote successful aging (Fiocco & Yaffe, 2010; Friedman & Ryff, 2012). Last but not least, aging is a dynamic concept, defined in terms of change in social roles and capabilities (Kowal & Dowd, 2001).

The current life expectancy at birth in New Zealand for men is 80.5 years and 84.1 years for women (Statistics New Zealand, 2022). Based on significant gains from net migration, increasing numbers and population at old age, and the gap between the number of births and deaths, the population of people aged 65 and above in New Zealand is projected to reach about 1.42 million in 2043, and 2.06 million in 2068 (Statistics New Zealand, 2016). Even though not everyone may age equally, it is important to maximize the time people live in a successful state of aging. Hence, identifying the determinants of successful aging is important in promoting successful aging among older adults. The transition of adults into old age and their trajectory of successful aging over time is important to gain an understanding of the trend in successful aging. We have addressed these questions from a nationally representative longitudinal survey of older adults in New Zealand with the pattern of successful aging on a continuum.

Identifying factors that may positively influence successful aging and how these factors influence aging in the long term have been of great interest to individuals and societies. Based on low mortality and low fertility, it is projected that by the year 2050, the population of older adults aged 65 years globally will be more than twice the number of children under the age of five and about the same as the number of children under the age of 12 (United Nations, Department of Economic and Social Affairs, Population Division, 2022). This year 2018 marked the first time in human history when the global population of older adults aged 65 years and over exceeded that of children under 5 years of age (United Nations, Department of Economic and Social Affairs, Population Division, 2019). This was because scientific, technological, and social advances continue to increase life expectancy in many countries, making older adults the fastest-growing part of the population (Swindell, 2012).

Successful aging was first used to explain maximal life satisfaction (Havighurst, 1961). Rowe and Kahn’s model is considered the most widely used perspective for successful aging (Bowling & Dieppe, 2005) and has been replicated by many researchers (Rowe & Kahn, 2015). Successful aging has often been misconstrued as longevity. A review of 18 studies consisting of 9360 adults aged 60 years and over suggested that older adults do not consider longevity as a primary component of successful aging (Bhattacharyya et al., 2022). A review of the literature on successful aging also found that centenarians did not meet the criteria for successful aging suggesting the longer one lives, the more difficult it is to meet the successful aging criteria (Robine, 2019). In contrast to Rowe and Kahn’s objective criteria for successful aging, an in-depth interview of 97 community-dwelling Chinese older adults aged 80 years and over reported that older adults define successful aging as being self-reliant, engaging in physical activity, maintaining financial security, staying connected in the community, and accepting reality (Chen et al., 2020).

Even though there are many studies on the prevalence and correlates of successful aging, there are a few detailed analyses of how these correlates affect successful aging in the long term. Also, no studies in New Zealand have explained aging using Rowe and Kahn’s model of successful aging or even attempted to expand on the model despite being the first aging model to have emanated from an interdisciplinary collaboration on aging. As such, it has been impossible to compare successful aging among older adults in New Zealand with other Organization for Economic Co-operation and Development (OECD) countries. Understanding successful aging among Māori, as indigenous people of New Zealand, is also important as there may be other factors of importance that explain aging among them. The World Health Organization defines health as “a state of complete physical, mental and social wellbeing and not merely the absence of disease or infirmity” (World Health Organization, 2020). Health, however, does not always remain as a “state” fixed to a particular point in time but rather changes over time. Just like health, successful aging is also not a static phenomenon but rather is on a continuum. As successful aging is on a continuum, it can be best assessed through data obtained from longitudinal studies. In this study, we defined successful aging as having no multimorbidity, high functional capacity, active life engagement (Rowe & Kahn, 1987), and expanded the definition by including good health-related quality of life. We also examined the trajectories of successful aging among older adults in New Zealand.

## Methods

### Data Source

The New Zealand Health, Work, and Retirement Longitudinal (NZHWR) Study sampled adults aged 55 years and over living in Aotearoa New Zealand. The study investigated aging within the broad areas of health and wellbeing, economic participation, and social participation. The biennial study began in 2006 by the Health and Aging Research Team (HART), School of Psychology, Massey University (Stephens et al., 2018). Data from this ongoing longitudinal study were suitable to answer our research questions and were accessed.

At baseline in 2006, New Zealand citizens and permanent residents aged 55–70 years were identified from the Electoral Roll. Electoral registration is compulsory for New Zealand citizens and permanent residents aged 18 years and over. To ensure their inclusion and representativeness, Māori were oversampled based on an attrition rate of 25% across four subsequent waves of data collection (Towers, 2006). Details about the NZHWR study have been previously published (Alpass et al., 2007). Briefly, older adults in institutions (prisons, nursing homes, and care centers) were excluded from the NZHWR study. A brief pre-notice letter was sent to the selected random sample from the electoral roll inviting them to participate in the NZHWR study, followed by a questionnaire and a free-post return envelope. A postcard was later sent to everyone in the sample, thanking those who had returned the questionnaire and encouraging those who had not responded. This process was repeated every 2 years to re-survey existing cohorts if they had not withdrawn from the study, were not deceased, or lost to contact. Completed questionnaires were received from 6662 participants at baseline (2006). At the second wave (2008), 2473 out of the initial sample of 6662 participated. The sample decreased to 1985 in 2010, 1865 in 2012, 1688 in 2014, 1563 in 2016, and 1433 in 2018. We analyzed de-identified data from 1433 older adults recruited at baseline of the NZHWR study in 2006 and followed up for 12 years through seven waves of data collection.

### Measures and Description of the Instrument

The data collection instrument at baseline included 92 questions divided into seven sections. Demographic characteristics included in this study are age, gender, ethnicity, marital status, educational level, and employment status. Disease conditions assessed were arthritis or rheumatism, cancer, diabetes, heart disease, hypertension, respiratory disease, and stroke. Questions included whether a doctor, nurse or other healthcare worker had informed the participants that they have each of the seven disease conditions. The response to these questions was either yes or no and coded 1 or 0, respectively. Having zero or one disease condition was coded as 1 and having two or more (multimorbidity) was coded as 0. Functional capacity was assessed using physical functioning (PF) and role emotional (RE) adapted from the 12-Item Short Form Health Survey (SF-12) questionnaire. The raw scores were used as an alternative scoring procedure (Hagell et al., 2017). Functional capacity was scored as 2 if a respondent has ≥ the median PF and RE scores at baseline, 1 if a respondent has ≥ the median PF or RE score at baseline, and 0 if a respondent has less than the median PF and RE scores at baseline. This categorization is known as the median splits approach (Decoster et al., 2011).

Engagement with life was assessed through participants’ involvement in childcare and caregiving. They were asked if they regularly provided unpaid care for their grandchildren or other people’s children, and if they provided care for someone with a long-term illness, disability, or frailty. These questions were adapted from the Australian Longitudinal Study on Women’s Health (Lee & Porteous, 2002). Engagement with life was scored 1 if the respondent was involved in childcare and/or caregiving, and zero if they were not involved in either. Health-related quality of life (HRQoL) was assessed with the Physical Component Summary (PCS) and Mental Component Summary (MCS) of the SF-12 instrument. The PCS and MCS scoring was done using the guidelines recommended by Ware et al. (1995) and Frieling et al. (2013). The PCS and MCS were scored using norm-based methods using the regression weights from the general New Zealand population estimated from the 2008 New Zealand General Social Survey data, consisting of 8721 adults. This involved computing standardized composite scores and scaling the scores to have a mean of 50 and a standard deviation of 10. Data was considered missing if a respondent fails to respond to one of the items and if there were out-of-range values. Higher scores indicated good physical and mental health. HRQoL was scored 2 if a respondent has ≥ the mean PCS and MCS norm-based scores at baseline, 1 if a respondent has ≥ the mean PCS or MCS norm-based score at baseline, and 0 if a respondent has less than the mean PCS and MCS norm-based score at baseline.

We used a meaningful grouping approach (Song et al., 2013) to compute successful aging composite scores by combining the scores from disease conditions, functional capacity, engagement with life, and HRQoL. The minimum and maximum successful aging composite scores were 0 and 6, respectively. For an older adult, having less than the median number of diseases is scored 1, more than the median physical functioning and role emotional scores are scored 2, ≥ the mean PCS and MCS norm-based scores are scored 2, and being engaged in childcare/caregiving is scored 1. This participant would have a successful aging composite score of six.

### Data Analysis

We conducted latent growth curve modeling (LGCM) to assess the intercept and slope of successful aging trajectories. The LGCM is an application of structural equation modeling for the analysis of repeated measures data. It is used in studying patterns and predictors of change over time in linear or nonlinear trajectories of behavior, attitudes, or other phenomena (Kwon & Mustillo, 2020). The intercept is the initial level of successful aging, and the slope is the rate of change in successful aging over time.

In this study, the time-dependent variables were measured at different time points, and the simple unconditional linear LGM can be described using the following equation

(1) $$y_{ti}=\eta_{0i}+\lambda_{t}\eta_{1i}+\epsilon_{ti}$$

where $y_{ti}$ is the i^th^ observed outcome (successful aging) at time t. The intercept and factor loading at a particular time t are represented by $\eta_{0i}$ and $\lambda_{t}$ , respectively. The intercept is the first latent factor in the model. It is a constant for any individual across time; hence, the factor loadings on the intercept growth factor are all fixed to 1.0. The factor loadings on the slope ( $\lambda_{t}$ ) were set at 0, 2, 4, 6, 8, 10, and 12 to indicate baseline and data collected at 2 years intervals. The slope factor represents the slope of an individual’s successful aging trajectory. $\eta_{1i}$ is the latent slope and $\epsilon_{ti}$ represents the residual term representing both random measurement error and time-specific influence of the i^th^ individual.

We used Multiple Indicators, Multiple Causes (MIMIC) models to examine the effect of baseline age, gender, and ethnicity on the growth factors. The baseline age was first included as a time-invariant factor in the model (Model 2) and the overall fit was examined. Gender (Model 3) and ethnicity (Model 4) were added to the model. Model fit indices were examined to make sure that the overall fit of the model did not decrease when the variables were added. We assessed measures of fit using the approximate fit indices. These are the relative/incremental fit indices such as the Comparative Fit Index (CFI) and Tucker Lewis Index (TLI). A CFI value ≥0.95 was considered a good fit while a TLI, also known as the Non-Normed Fit Index (NNFI) of ≥0.90 was considered a good fit. An absolute fit index such as the Root Mean Square Error of Approximation (RMSEA) was also used to evaluate the model fit. An RMSEA value <0.05 was considered a good model fit and an RMSEA within 0.05–0.08 was considered a reasonable fit. The Standardized Root Mean Square Residual (SRMR) which is the difference between the observed correlation and the model implied correlation matrix was also used to evaluate the model fit. Values within 0 and 0.08 are considered a good fit (Hu & Bentler, 1998).

We conducted statistical analyses using R (R Core Team, 2021). Path diagrams for the Structural Equation Models (SEM) were created using Ωnyx—a free software environment—and were estimated using the Lavaan package (Rosseel, 2012). We handled missing data using the Full Information Maximum Likelihood (FIML) estimation (Burant, 2016). The Human Ethics Review for this study was the same as the original one shared by the Health and Aging Research Team, Massey University (PN320/PN601).

## Results

The majority of the participants were females (54.6%), and non-Māori (66.0%) (Table 1), and the mean age and standard deviation at baseline were 60.8 years and 4.4 years, respectively.

**Table 1.** Characteristics of the Participant at the Study Baseline (2006).

Characteristics	*N*	Percent
Gender		
Male	651	45.4
Female	782	54.6
Ethnicity		
NZ European	879	61.3
Māori	487	34.0
Pacific Peoples	7	0.5
Asian	2	0.1
Other	58	4.1
Age group (years)		
≤59	638	44.5
60–64	465	32.5
65 and over	330	23.0
Marital status		
Married or de facto	1105	77.1
Not married or de facto	316	22.1
Missing data	12	0.8
Educational level		
No qualifications	375	26.2
Secondary	333	23.2
Tertiary	704	49.2
Missing data	21	1.4
Employment status		
Working	1006	70.2
Retired	265	18.5
Unemployed	12	0.8
Other^a^	117	8.2
Missing data	33	2.3

^a^full-time homemaker, full-time student, unable to work due to health or disability issue, and undefined work status.

The mean (standard deviation) successful aging score decreased from 3.64 (SD = 1.64, 25th percentile = 2.00, median = 4.00, 75th percentile = 5.00) at baseline to 2.78 (SD = 1.74, 25th percentile = 1.00, median = 3.00, 75th percentile = 4.00) in the seventh wave. Table 2 shows that the mean successful aging score was higher among males than females from the first wave to the seventh wave. Non-Māori had higher mean successful aging scores than Māori across the study waves. There was a decline in the successful aging mean score across the age groups. However, the largest drop (1.31 units) was among those aged 65 years and over at baseline compared with 0.64 units among those who were less than 60 years at baseline.

**Table 2.** Descriptive Statistics for Successful Aging Score Across the Study Waves (2006–2018) by Gender, Ethnicity, and Age Group. The Numbers Represent the Averaged Successful Aging Score and Standard Deviations are Presented in the Parentheses (*N* = 1433).

	2006	2008	2010	2012	2014	2016	2018
	Mean (SD)	Mean (SD)	Mean (SD)	Mean (SD)	Mean (SD)	Mean (SD)	Mean (SD)
Overall	3.64 (1.69)	3.33 (1.74)	3.12 (1.77)	3.16 (1.71)	2.97 (1.75)	2.93 (1.71)	2.78 (1.74)
Gender							
Male	3.76 (1.68)	3.40 (1.71)	3.27 (1.77)	3.25 (1.72)	3.04 (1.78)	3.00 (1.74)	2.83 (1.75)
Female	3.55 (1.70)	3.27 (1.77)	3.01 (1.75)	3.08 (1.70)	2.90 (1.72)	2.87 (1.69)	2.74 (1.74)
Ethnicity							
Māori	3.35 (1.68)	3.13 (1.76)	2.79 (1.77)	2.85 (1.70)	2.80 (1.72)	2.71 (1.71)	2.53 (1.72)
Non-Māori	3.80 (1.68)	3.43 (1.73)	3.30 (1.74)	3.32 (1.69)	3.05 (1.75)	3.05 (1.71)	2.91 (1.72)
Age group (years)							
≤59	3.76 (1.68)	3.45 (1.75)	3.22 (1.78)	3.28 (1.73)	3.19 (1.77)	3.19 (1.75)	3.12 (1.76)
60–64	3.59 (1.69)	3.34 (1.70)	3.11 (1.72)	3.20 (1.67)	2.98 (1.70)	2.94 (1.65)	2.73 (1.66)
65 and over	3.50 (1.69)	3.09 (1.78)	2.95 (1.79)	2.86 (1.67)	2.52 (1.68)	2.43 (1.62)	2.19 (1.66)

The average successful aging baseline (mean latent intercept) score was lower among Māori compared with non-Māori. For every sequential wave, the successful aging score for all the participants decreased. The highest significant linear decrease (−0.11) was observed among non-Māori females aged ≥65 years.

**Figure 1.**
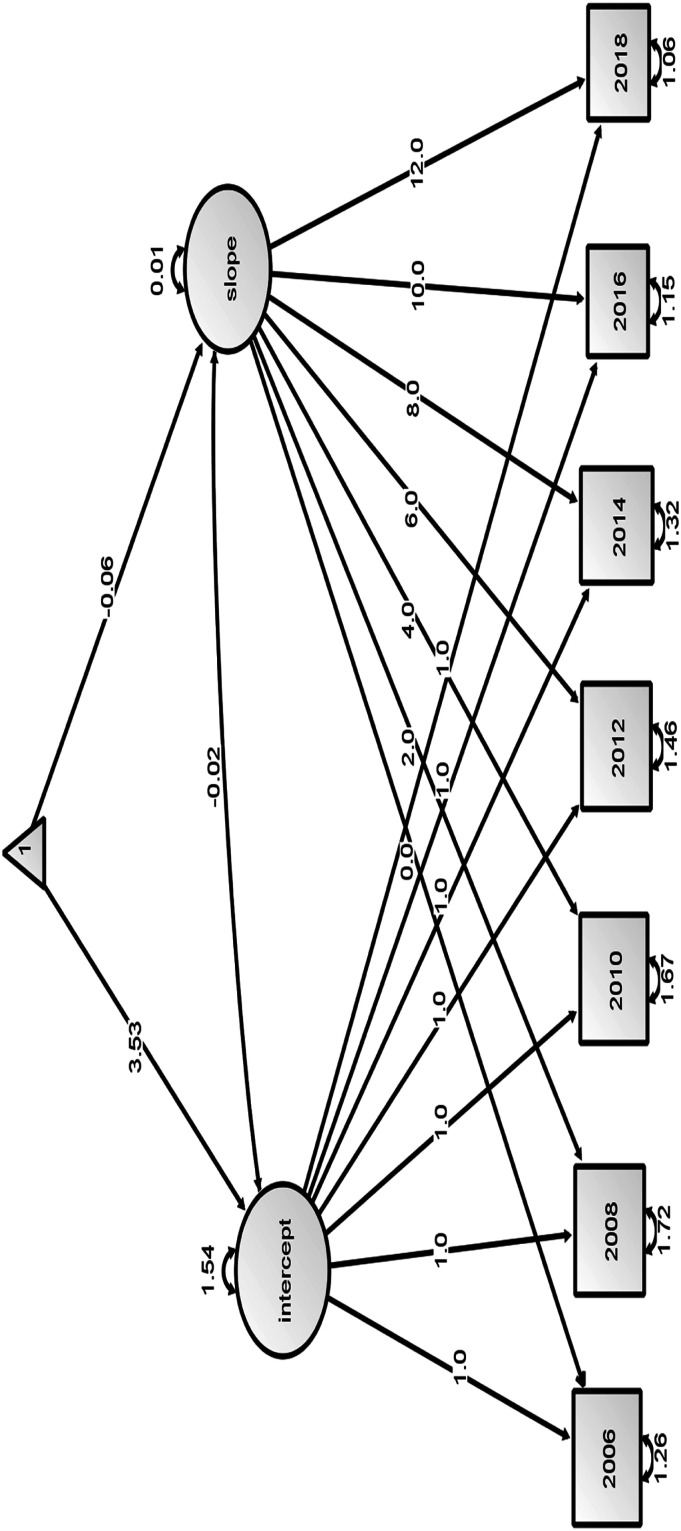
Model 1—Unconditional Latent Growth Model for Seven Repeated Measures of Successful Aging. The Coefficients of the Intercepts are Fixed at 1.0 and Those From the Slope to the Repeated Measures Took the Values of 0.0 (Baseline), 2.0 (Second Wave), 4.0 (Third Wave), 6.0 (Fourth Wave), 8.0 (Fifth Wave), 10.0 (Sixth Wave), and 12.0 (Seventh Wave). Successful Aging Scores for the Seven Waves are 2006–2018. The Triangle Represents Mean Score, One-Way Arrows Represent Paths, and Two-Way Arrows Represent Variances or Covariances. The Mean Intercept Score of Successful Aging was 3.53, the Slope was −0.064, and Covariance Between the Slope and Intercept was −0.02.

Assuming linear growth, the mean intercept score of successful aging was 3.53 and the slope was −0.064 (Figure 1). This indicates that on average, there was a linear drop of 0.064 units in the successful aging score for all participants in the study. The slope was negatively correlated with the intercept indicating that those who had higher baseline scores had a slower rate of decline (correlation = −0.02, *p* < 0.001).

Tables 3 and 4 show the MIMIC model and the effect of gender, baseline age, and ethnicity on the intercept and slope factors. Male gender, age group less than 60 years, and non-Māori were used as reference groups. On average, there was a significant linear decrease in the successful aging score across the study waves. The regression coefficient of the effect of gender, age, and ethnicity on the intercept factor shows that the mean scores were statistically lower among females than males, 65 years and over than those less than 60 years, and among Māori than non-Māori. The regression estimate from age to the intercept was negative, which implies that those aged 60 years and over had a lower initial successful aging score than those aged less than 60 years. Those aged 65 years and over also exhibit a significant linear decrease in their successful aging scores than those aged less than 60 years across the waves.

**Table 3.** Parameter Estimates for the Conditional Latent Growth Model of Successful Aging (Intercept).

	*N*	%	Model 2	Model 3	Model 4
			Estimate (SE)	Estimate (SE)	Estimate (SE)
Intercept			3.86*** (0.13)	3.94*** (0.14)	4.48*** (0.17)
Gender					
Male	651	45.4	Ref	Ref	Ref
Female	782	54.6	−0.22** (0.08)	−0.22** (0.08)	−0.19* (0.08)
Age (years)					
≤59	638	44.5		Ref	Ref
60–64	465	32.5		−0.08 (0.09)	−0.10 (0.09)
65 years and over	330	23.0		−0.19 (0.10)	−0.22* (0.10)
Ethnicity					
Non-Māori	946	66.0			Ref
Māori	487	34.0			−0.43*** (0.08)
CFI			0.978	0.978	0.977
TLI			0.978	0.976	0.973
SRMR			0.032	0.030	0.025
RMSEA (95% CI)			0.048 (0.039–0.057)	0.041 (0.033–0.049)	0.040 (0.032–0.047)

Gender was added and retained as a predictor in Model 2, baseline age was added and retained in Model 3, ethnicity was added and retained in Model 4, ***Significant at 0.001, **Significant at 0.01, *Significant at 0.05, RMSEA = Root Means Square Error of Approximation.

**Table 4.** Parameter Estimates for the Conditional Latent Growth Model of Successful Aging (Slope).

	*N*	%	Model 2	Model 3	Model 4
			Estimate (SE)	Estimate (SE)	Estimate (SE)
Slope			−0.08*** (0.01)	−0.06*** (0.01)	−0.06*** (0.02)
Gender					
Male	651	45.4	Ref	Ref	Ref
Female	782	54.6	0.01 (0.01)	0.01 (0.01)	0.01 (0.01)
Age (years)					
≤59	638	44.5		Ref	Ref
60–64	465	32.5		−0.02* (0.01)	−0.02 (0.01)
65 years and over	330	23.0		−0.06*** (0.01)	−0.06*** (0.01)
Ethnicity					
Non-Māori	946	66.0			Ref
Māori	487	34.0			0.01 (0.01)
CFI			0.978	0.978	0.977
TLI			0.978	0.976	0.973
SRMR			0.032	0.030	0.025
RMSEA (95% CI)			0.048 (0.039–0.057)	0.041 (0.033–0.049)	0.040 (0.032–0.047)

Gender was added and retained as a predictor in Model 2, baseline age was added and retained in Model 3, ethnicity was added and retained in Model 4, ***Significant at 0.001, **Significant at 0.01, *Significant at 0.05, RMSEA = Root Means Square Error of Approximation.

## Discussion

Our study is the first one in New Zealand to examine the trajectory of successful aging with Rowe and Kahn’s model of successful aging despite its wide use. The mean successful aging score declined from 3.64 in 2006 to 2.78 in 2018. The highest mean score was reported among those ≤59 years at baseline, males, and non-Māori. At baseline, the LGCM shows that the successful aging scores of females were lower than that of males, and females exhibit a steeper drop than males as age increases. The mean successful aging scores among Māori were statistically lower at baseline and exhibit a steeper drop compared with non-Māori. Age, gender, and ethnicity were significantly associated with the intercept factor. However, only age was significantly associated with the slope factor.

### Successful Aging and Age

We found that the lower age group had higher scores for successful aging across the study waves, suggesting that numerical age is a determinant of the aging process itself. A longitudinal study from Sweden on the association between life-course working conditions and successful aging in later life also reported that those in the youngest age group had better successful aging scores compared to those in the oldest age group (Nilsen et al., 2022). A survey of Mediterranean older adults aged 65 years and over has reported that successful aging scores decrease with age and the greatest decline was among those who were 80 years or over compared to those who were in the 65–80 years age group (Tyrovolas et al., 2018). A longitudinal study of 12,432 women from Australia has found that those who are in their early 70s were more likely to be successful agers compared to those in their 80s (Byles et al., 2019). Studies have been consistent in reporting a negative association between age and successful aging which agrees with the findings of our study.

### Successful Aging and Gender

Research reports on the effects of gender on successful aging have been inconsistent. We have found that the majority of men in the sample had higher scores than women across the study waves. A cross-sectional survey of 370 older adults aged 65 years and over in Turkey reported a higher mean successful aging score among males (*p* = 0.258) (Yalcinoz Baysal et al., 2020). Depp and Jeste’s (2009) meta-analysis of predictors of successful aging found that women were more likely to experience successful aging than men. A longitudinal study among 674 Swedish older adults reported that women had higher mean successful aging scores (2.1 ± 1.4) than men (1.9 ± 1.4) (Nilsen et al., 2022). The probable explanation for the inconsistencies in these reports could lie in the participants sampled, differences in the methods used, and measurement of successful aging. Stewart et al. (2018) in their data analysis of older adults aged 65–75 years living in Canada, Columbia, Brazil, and Albania reported that for every one-point increase in the number of chronic conditions, the odds of successful aging decreases by 14% (*p* = 0.013). We also found that multimorbidity was higher among females which could be one of the reasons females had lower successful aging scores compared with their male counterparts. The current life expectancy at birth in New Zealand shows that women are living longer than men. However, our findings suggest that men experienced better quality of life than women as they aged.

### Successful Aging and Ethnicity

The successful aging mean score was higher among non-Māori compared to Māori across the study waves. Across the study waves, Māori had statistically significantly lower successful aging mean scores than non-Māori. Indigenous peoples have been reported to have a shorter life span, have a disproportionately high risk of developing chronic diseases, and are more likely to have multiple comorbidities than their mainstream counterparts (Goins & Pilkerton, 2010; Jacklin et al., 2013; Wilson et al., 2010). Māori are the indigenous population of New Zealand and have a lower life expectancy at birth than non-Māori (Parr-Brownlie et al., 2020). The successful aging mean score reported in this study is in contrast to the findings among 800 Chilean older adults where the successful aging mean score was higher among indigenous people compared to non-indigenous Chileans. The higher successful aging mean score among these Chilean older adults was attributed to cultural factors such as the upholding of cultural traditions that promote a healthy and natural lifestyle (Gallardo-Peralta et al., 2022). A literature review by Pace and Grenier (2016) suggests that the consideration of inequality may be a key to creating successful aging models that are inclusive and consider diverse groups of older adults. The creation of the Māori Health Authority in July 2022 is expected to address some of the health inequalities faced by the indigenous people of Aotearoa New Zealand, including older Māori adults.

Being based on a longitudinal study, the data allowed us to assess the relationship between successful aging and time-invariant variables using a latent growth modeling approach. The multivariate technique allowed the temporal relationships among key predictors and the outcome of interest to be established. The strength is further evidenced by using a large cohort of community-dwelling older adults who were 65 years or over at the seventh wave and were followed for 12 years. However, self-reported data were available in this study to define successful aging and to measure variables in the model. Self-reported health has been shown to be a valid and reliable measure of overall health (Ferdows et al., 2018).

### Implications

As people age, it is important that they age successfully. Even though not everyone will age equally, it is nevertheless important to minimize the time people live in a less than successful state of aging. Avoidance of multimorbidity, high functional capacity, active engagement with life, and good health-related quality of life are important factors that can help older adults age successfully. However, this needs to be achieved and maintained across the life course. Since our findings revealed that those who had higher successful aging scores at baseline had a slower decline across the study waves, it is important to prepare for and improve on the health and wellbeing of New Zealand adults as they transition into old age.

The population of older people in New Zealand is increasing and becoming more diverse with rising proportions of Māori, Pacific Peoples, and Asian populations. This diversity and population increase will change the cultural make-up of New Zealand. This will result in a need for an introduction of a range of different approaches and support for older adults. Services for older adults have generally focused on New Zealand European needs. This has led to the marginalization of other minority ethnic groups thereby affecting how they successfully age. There are existing barriers limiting Māori and other ethnic minorities from accessing healthcare and other services, and these barriers need to be addressed to promote successful aging. This can be achieved through research and changes in policies throughout the socioeconomic, health and wellbeing, justice, and education sectors (Parr-Brownlie et al., 2020). Te Whatu Ora/Health New Zealand and Te Aka Whai Ora/Māori Health Authority established in 2021 could also focus on delivering quality healthcare services to Māori and other minority ethnic groups.

A Kaupapa Māori health research approach which promotes structural analysis of Māori health disparities that moves the discourse away from victim-blaming and personal deficits to understanding people’s lives and the systemic determinants of health and wellness needs to be employed (Cram, 2019). Longitudinal studies can identify the determinants and predictors of successful aging. Identifying these factors and the roles they play in successful aging and how they differ among individuals and various ethnic groups in New Zealand can inform policy makers and help make the changes required.

### Limitations

Our study is not without limitations. First, it is possible that there are time-varying covariates that have significant impacts on how older adults successfully age. These were not included in our models. As an analysis of secondary data, this study was limited to available variables. Also, it was not clear how many participants among the baseline sample died, relocated, or declined further participation in the study and if the demographic characteristics of those who dropped out differ from those who were retained in the study. The small sample of Pacific Peoples, Asian, Middle Eastern, Latin American, and African participants in this study limits the generalizability of our findings to these ethnic groups in New Zealand.

## Conclusions

Successful aging is on a continuum and can be best understood through longitudinal studies. Avoidance of multimorbidity, good functional capacity, active life engagement, and good health-related quality of life are important determinants of successful aging. Young older adults, males, and non-Māori were observed to experience successful aging more. This suggests the possibility of gender and ethnic inequalities in the factors that promote successful aging among older adults in New Zealand. Social and health reforms that would address these inequalities are needed in New Zealand.
